# Haemoptysis in a female with diabetes mellitus: a unique presentation of chronic pulmonary aspergillosis, pulmonary tuberculosis, and *Klebsiella peumoniae* co‐infection

**DOI:** 10.1002/ccr3.542

**Published:** 2016-03-17

**Authors:** Chinonso Ekwueme, Akaninyene Asuquo Otu, Sunny Chinenye, Chioma Unachukwu, Reginald N. Oputa, Ibitrokoemi Korubo, Ofem E. Enang

**Affiliations:** ^1^ Endocrine UnitDepartment of Internal MedicineUniversity of Port Harcourt Teaching HospitalPort HarcourtRivers StateNigeria; ^2^ Department of Internal MedicineUniversity of CalabarCalabarCross River StateNigeria; ^3^ Endocrine UnitFederal Medical CentreOwerriImo StateNigeria

**Keywords:** Chronic pulmonary aspergillosis, diabetes mellitus, *Klebsiella pneumonia*, pulmonary tuberculosis

## Abstract

While chronic pulmonary aspergillosis (CPA), pulmonary tuberculosis (PTB), and *Klebsiella pneumoniae* pneumonia co‐infection is rare, we present a 50‐year‐old woman with uncontrolled diabetes who presented with these three diseases. There is considerable overlap in symptoms of PTB and CPA. Treatment with antifungals, anti‐tuberculosis therapy, and antibiotics is beneficial.

## Introduction

Diabetes mellitus is metabolic disorder of multiple etiologies with profound negative effects on neutrophil proliferation, maturation, function, and lifespan 1. This predisposes individuals with diabetes to a wide range of opportunistic infections such as pulmonary tuberculosis (PTB). Some hypotheses suggested to explain this phenomenon of diabetes mellitus acting as a predisposing risk factor for PTB include: depressed cellular immunity, dysfunction of alveolar macrophages, low levels of interferon gamma, pulmonary microangiopathy, and micronutrient deficiency 2, 3.

Aspergillus is a ubiquitous fungus that causes a spectrum of pulmonary diseases ranging from a noninvasive disease to an invasive infection. Unlike the invasive aspergillosis, chronic pulmonary aspergillosis (CPA) occurs in immunocompetent patients 4. CPA is also reported to be the most subtle, yet severe long‐term complication of PTB that is probably more common than is generally appreciated 4. It can account for progressive lung destruction and the persistence of symptoms after successful anti‐tuberculous treatment and can mimic smear‐negative PTB 4. There is considerable overlap in symptoms of PTB and CPA with haemoptysis, weight loss, fatigue, and shortness of breath being common features.

In this case report, we present PTB, CPA, and Klebsiella pneumonia co‐infection presenting as haemoptysis in a female with uncontrolled diabetes mellitus. This combination is rare and to the best of our knowledge not reported in literature.

## Case History/Examination

A 56‐year‐old Nigerian woman was referred to the medical outpatient department of a tertiary hospital in Southern Nigeria with complaints of productive cough and haemoptysis of 2 weeks duration. She also complained of anorexia, weight loss, low‐grade fever, and extreme fatigue. There was no history of contact with anyone who had a chronic cough and she had never smoked.

About 6 months prior to presentation, she was diagnosed with PTB and coexisting diabetes mellitus. Her father had diabetes mellitus, but none of her siblings were affected. She was started on insulin and anti‐tuberculous therapy consisting of rifampicin, isoniazid, ethambutol, and pyrazinamide. Two months into her anti‐tuberculosis therapy, she discontinued treatment when she began to feel much better.

General examination revealed pallor and a body mass index of 24.1 kg/m^2^. Respiratory examination revealed tachypnea with left infraclavicular bronchial breath sounds and coarse crackles in the left lung base.

## Differential Diagnosis, Investigations, and Treatment

**Figure 1:**
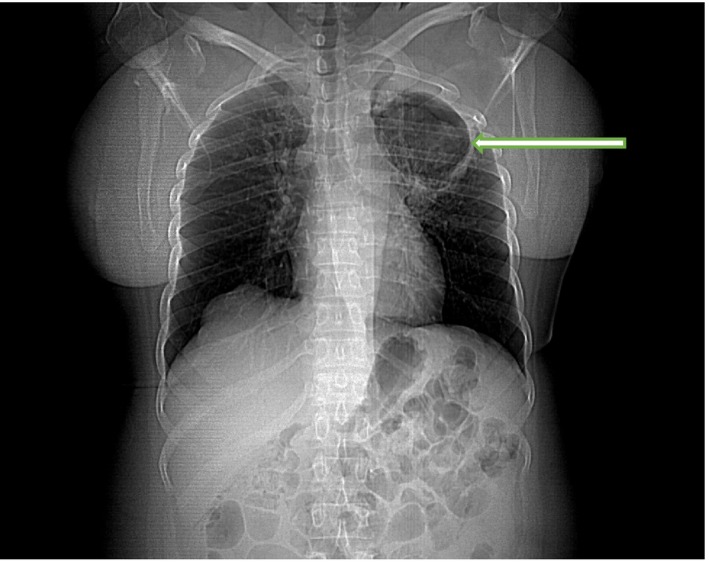
Chest CT scan showing a thick‐walled cavity in the left upper lobe.

**Figure 2:**
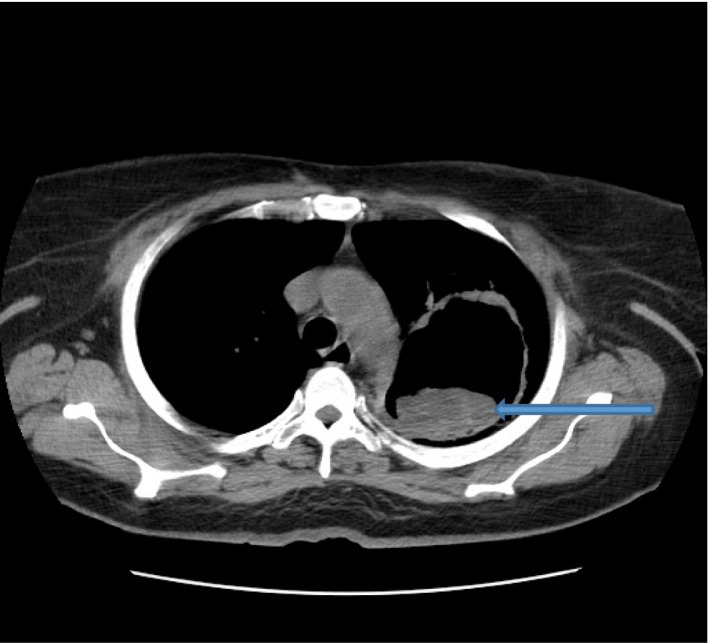
Chest CT scan showing a cavity with a hyperdense shadow in the dependent area.

Her chest radiograph showed a large thick‐walled cavity in the left upper lung zone containing a solitary oval‐shaped opacity. Ziehl‐Neelsen staining of the sputum was negative for acid fast bacilli. However, Cepheid Xpert^®^ MTB/RIF assay of the sputum detected very low levels of *Mycobacterium tuberculosis* which was not resistant to rifampicin. Sputum microscopy using potassium hydroxide identified some fungal elements, however, no fungi were isolated after 2 weeks of culture. The Aspergillus precipitin test yielded high *Aspergillus fumigatus* immunoglobulin G (IgG) levels of 172 mg/dL (Table 1). Sputum bacterial culture also yielded moderate growth of *Klebsiella pneumoniae*. A chest computed tomography (CT) scan showed a thick wall cavity measuring 8.4 × 6.4 × 6.7 cm in the left upper lobe with a nodular hyperdense mass within in a dependent position (Figs. 1, 2). Her random blood glucose on admission was 15 mmol/L with a HbA1c of 11.5%. Other blood biochemical parameters are shown in Table 1.

**Table 1:** Laboratory investigations

Hemoglobin	8.0 g/L
Total white blood cell count	10.6 × 10^9^/L
Neutrophils	69.7%
Lymphocytes	20.6%
Monocytes	8.3%
Eosinophils	1.0%
Basophils	0.4%
Platelets	456 × 10^9^/L
Erythrocyte sedimentation rate (ESR)	120 mm/h
Serum urea	1.3 mmol/L
Serum creatinine	60 μmol/L
eGFR	113 mL/min/1.73 m^2^
Serum bilirubin	5 μmol/L
Serum protein	83 g/dL
Serum albumin	40 g/L
ELISA for HIV 1 and 2	Non‐reactive
HBsAg	Negative
Anti‐HCV	Negative
Haemoglobin A1c	11.5% (<6.5%)
Random glucose on presentation	15 mmol/L
Sputum AFB (3 samples)	Negative
GeneXpert	Low *Mycobacterium tuberculosis* detected Rifampicin resistance not detected
Sputum bacterial culture	Moderate *Klebsiella pneumoniae*
Sputum fungus microscopy (KOH)	Moderate fungal elements
Aspergillus precipitin test	
*Aspergillus fumigatus* IgG (ImmunoCAP)	172 mg/L (<40 mg/L)

She was initially treated with daily doses of rifampicin (600 mg), isoniazid (300 mg), ethambutol (800 mg), pyrazinamide (1 g), and pyridoxine (50 mg). Following the detection of Aspergillosis and *Klebsiella pneumonia*, she was started on itraconazole 200 mg 12 hourly for 6 months and ceftazidime 1 g given 12 hourly for 10 days. Her hyperglycemia was treated with a combination of soluble insulin and insulin glargine.

## Outcome and Follow‐up

Her haemoptysis stopped within a week of treatment and the cough gradually subsided. Her appetite improved considerably with a concomitant increase in weight and body mass index to 26 kg/m^2^. The fever abated and she was able to perform all activities of daily living with an overall improvement in glycemic control. She is currently in the second month of follow‐up.

## Discussion

Diabetes mellitus continues to be a global health problem with over 80% of affected people living in low‐middle income countries (LMICs) where tuberculosis is widespread 5. Nigeria is ranked fifth among the 22 nations with high tuberculosis burden 6 with 94,825 new and relapsed cases of tuberculosis recorded in 2013 7. Recent studies from Nigeria have reported an increasing frequency of diabetes among persons with tuberculosis 8, 9, 10. Diabetes mellitus causes immunosuppression which is linked to dysfunction of granulocyte adherence, chemotaxis, and phagocytosis with associated myeloperoxidase deficiency and complement pathway defect 11. Diabetes mellitus is a major growing risk factor for pulmonary infections with *Mycobacterium tuberculosis* 12 and *Klebsiella pneumoniae* 13 both of which our patient had. *Aspergillus fumigatus* rarely causes disease in humans, but CPA can commonly occur in the setting of predisposing diseases such as classical and atypical tuberculosis, chronic obstructive pulmonary disease (COPD)/emphysema, pneumonia, pneumothorax, and previous thoracic surgery 14, 15.

The term CPA refers to a group of specific entities which usually present with chronic cough, dyspnea and weight loss, and occasional acute symptoms such as haemoptysis 16. These symptoms are very difficult to distinguish from those of pulmonary infections with *Mycobacterium tuberculosis* and *Klebsiella pneumoniae*. The different patterns of CPA are *Aspergillus* nodule, single (simple) aspergilloma, chronic cavitary pulmonary aspergillosis, and chronic fibrosing aspergillosis. These entities tend to run an indolent clinical course with progressive destruction of the lungs with cavity formation the most common radiological feature 17. Our patient's chest CT scan revealed a mobile and dependent opaque mass within the cavity that is characteristic of aspergillomas. Important to the diagnosis of CPA is the identification of serologic Aspergillus IgG antibodies as seen in the index patient. Serology tests for Aspergillus *fumigatus* IgG antibody have a sensitivity of 90% for CPA 17.

Chronic pulmonary aspergillosis can complicate tuberculosis of the lungs especially when there is pulmonary cavitation which is the hallmark of established PTB and this is often associated with high bacillary load 18. However, very low *Mycobacterium tuberculosis* bacilli levels were detected in our patient who had a pulmonary cavity. The prevalence of CPA as a sequel of PTB varies worldwide. Nigeria has a 5‐year estimated prevalence of 42.9% which is second highest globally 2. This highlights the need to consider testing for CPA in patients with tuberculous cavities especially if they are not improving on anti‐tuberculous therapy. Incorporation of CPA testing into national tuberculosis control program algorithms is likely to enhance the identification of cases of CPA among persons with PTB.

Sputum smear microscopy for acid fast bacilli remains the most widely used test for PTB 19. However, this test has a sensitivity of only 50% which is even lower in the setting of human immunodeficiency virus (HIV) infection. The Xpert assay (Cepheid, Sunnyvale, CA) is a fully automated real‐time DNA‐based test which has high sensitivity in detection of smear‐negative TB. Xpert has the added advantage of detecting both TB and rifampicin resistance 20 and provides results in a little as 2 h. This hemi‐nested PCR test potentially decreases the default due to delayed diagnosis 21 which is commonly encountered in the evaluation of smear‐negative cases.

Antifungal agents such as voriconazole, itraconazole, and liposomal amphotericin B are useful in treating CPA although azole resistance is of increasing concern. It is important to consider drug interactions when managing CPA and PTB co‐infection. Rifampicin causes significant cytochrome P450 induction, which may result in significant decreases in triazole serum concentrations and therapeutic failure 22, 23. In situations where rifampicin has to be co‐administered with triazole antifungals, the level of the triazole in serum needs to be monitored. Unfortunately, facilities for monitoring the serum level of itraconazole were not available for the index patient. Although surgery has a limited role to play in treatment of CPA, it is curative in limited disease such as patients with single(simple) aspergillomas. Surgical resection can also be done for those with significant haemoptysis 15. Embolization may be appropriate where disease is extensive or patients are not fit for surgery. Rifampicin, isoniazid, ethambutol, and pyrazinamide remain the first‐line drugs for the treatment of susceptible *Mycobacterium tuberculosis* strains. *Klebsiella pneumoniae* lung infection has a high mortality regardless of treatment 24. This infection is best treated with quinolones, carbapenems, aminoglycosides or third‐ and fourth‐generation cephalosporins 25.

## Conclusion

PTB, CPA, and *Klebsiella pneumoniae* lung infection can coexist in patients with diabetes mellitus. There is a considerable overlap in the clinical presentation of these serious pulmonary infections hence the need for a high index of suspicion, appropriate investigation, and treatment in relevant situations.

## Conflict of Interest

None declared.
